# Small Bowel Obstruction Secondary to Intussuscepted Meckel's Diverticulum in an Adult

**DOI:** 10.1155/2019/3241782

**Published:** 2019-11-29

**Authors:** Daniel John Marascia

**Affiliations:** Eastern Health, Level 4, 5 Arnold St., Box Hill, Melbourne, Victoria 3128, Australia

## Abstract

Intussusception secondary to inverted Meckel's diverticulum resulting in intestinal obstruction is rare. The following is a case report that discusses a 29-year-old female who posed diagnostic uncertainty for the treating surgical team and ultimately underwent emergency surgery for the management of intestinal obstruction. Small bowel intussusception was diagnosed preoperatively on abdominal computer tomography (CT). At operation, it was found to be secondary to inverted Meckel's diverticulum with histopathology confirming the diagnosis.

## 1. Introduction

Intussusception is the telescoping a proximal segment of the bowel within the lumen of the adjacent segment. The condition is frequently noted in paediatric population but is, however, less prevalent in adults, accounting for only 5% of cases. Intussusception secondary to inverted Meckel's diverticulum is rarer again, with only 4% of cases of intussusception that present with intestinal obstruction occur secondary to inverted Meckel's diverticulum.

Meckel's diverticulum is the most common congenital abnormality in the gastrointestinal tract. It is considered a true diverticula which arises from failure to obliterate the vitelline duct during embryonic development. When symptomatic, Meckel's diverticulum in adults will present with intestinal obstruction or bleeding and will require resection of the small bowel involved.

## 2. Case Presentation

A 29-year-old Caucasian female student presented to the Emergency Department with a four-day history of abdominal pain with associated vomiting, abdominal bloating, constipation, and anorexia. The onset of the abdominal pain occurred within hours following the first dose of NSAID prescribed for the management of a musculoskeletal complaint. The patient was systemically well. Relevant past history included gastro-oesophageal reflux disease. No regular medications, allergy to roxithromycin, and no significant family history. On examination, the patient appeared clinically hypovolaemic but haemodynamically stable and was afebrile. The abdomen appeared mildly distended, soft but with generalized tenderness in the absence of peritonism and the presence of normal bowel sounds.

**Figure 1:**
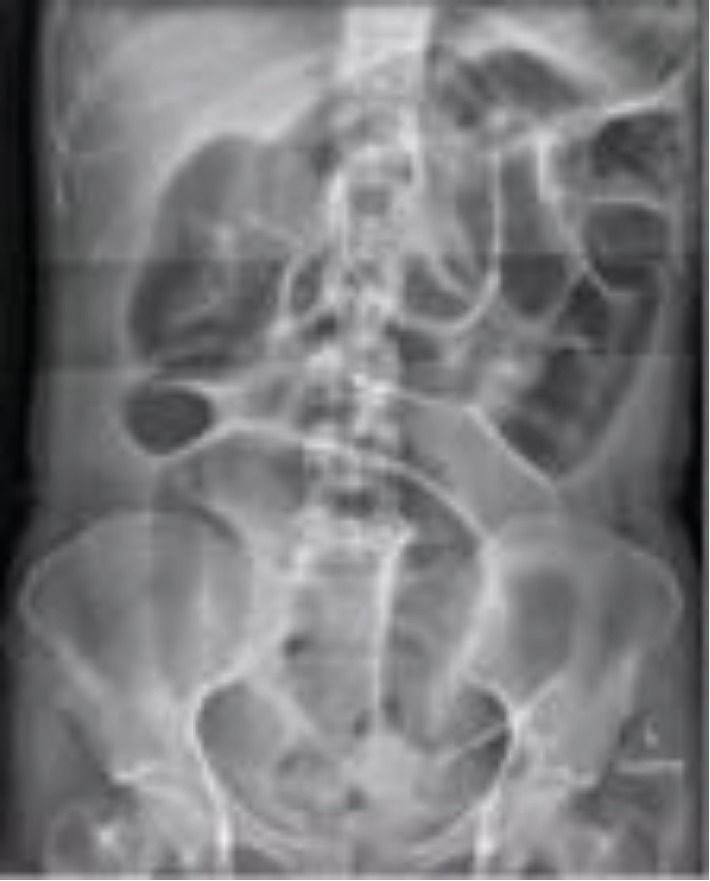
Abdominal X-ray image demonstrating multiple dilated loops of the small bowel.

Laboratory tests revealed mildly elevated white cells, a CRP of 37 mg/L (ref. range: <2 mg/L) and mildly raised lipase of 191 U/L (ref. range: 7-60 U/L). Liver function tests and electrolytes were normal. Abdominal X-ray (AXR) revealed diffuse distention of small bowel loops without evidence of free gas within the peritoneum (Figure 1). Initial differentials included peptic ulcer disease and gastritis, with the possibility of ileum versus small bowel obstruction (SBO) considered also. A proton pump inhibitor (PPI) infusion was commenced to good effect and an abdominal ultrasound was ordered demonstrating a mildly thickened and hyperaemic gallbladder wall with mobile sludge raising suspicion of acute cholecystitis. Intravenous antibiotics were commenced, and plan for cholecystectomy was made with the view that the dilated small bowel loops were likely in keeping with a reactive ileus.

**Figure 2:**
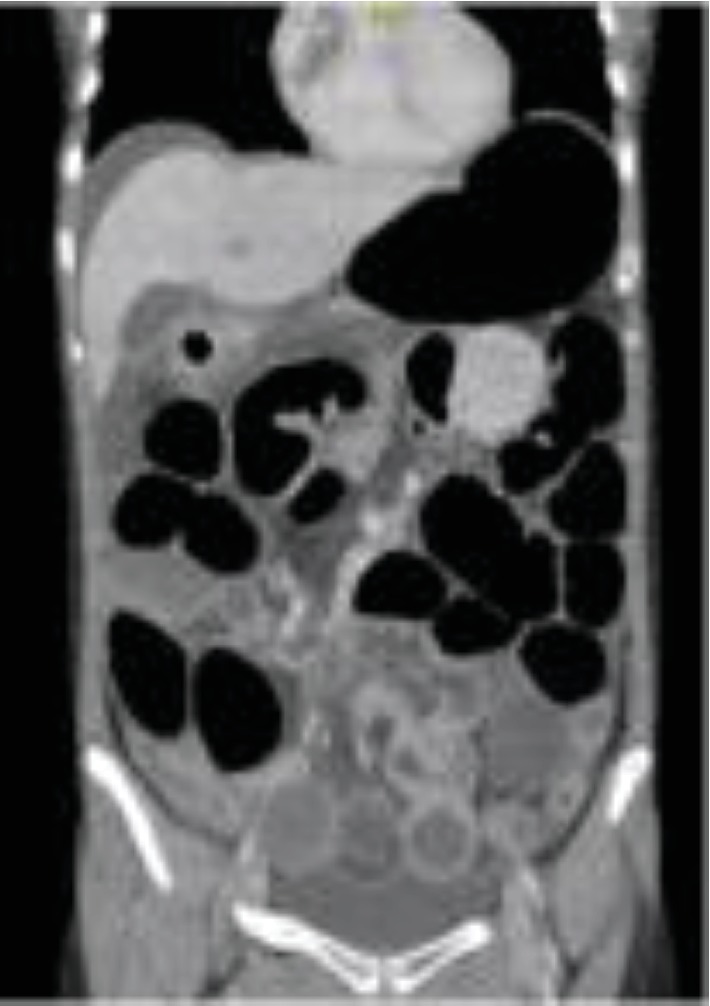
Computer tomography scan in the coronal plane demonstrating high-grade distal small bowel obstruction with a transition point within the left iliac fossa. Appearance of a “target” sign raising suspicion of an intussusception.

**Figure 3:**
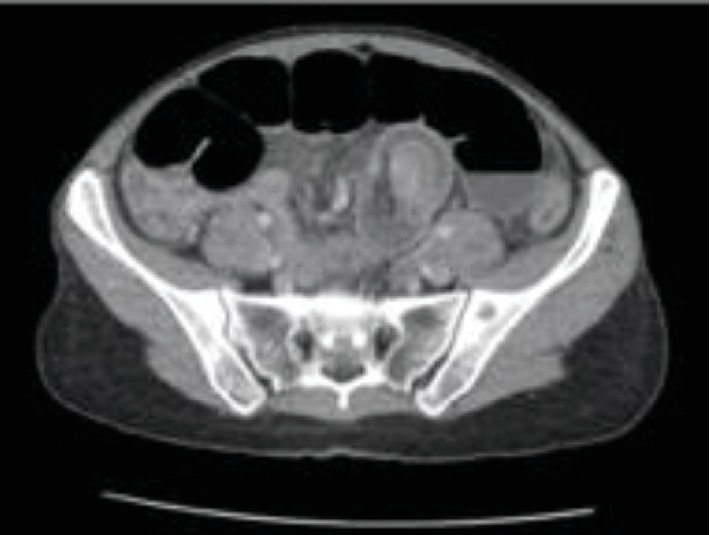
Computer tomography scan in the axial plane demonstrating multiple loops of dilated loops of the small bowel with and transition point within the left iliac fossa with the characteristic “target” sign suggesting high-grade small bowel obstruction likely secondary to intussusception.

Inflammatory markers continued to trend upwards, abdominal pain worsened, and constipation continued. Abdominal CT scan was ordered which revealed high-grade distal SBO with transition point in the left iliac fossa and signs suggestive of ileo-ileal intussusception (Figures 2 and 3). The patient was taken to theatre for diagnostic laparotomy where intussusception of small bowel secondary to inverted Meckel's diverticulum was diagnosed. A segmental resection of 15 cm of distal ileum 10 cm proximal to the caecum with a side-to-side anastomosis was performed. Histopathology of the resected specimen demonstrated Meckel's diverticulum with associated ulceration and inflammatory infiltrate secondary to the intussusception. The patient returned to the ward and had an uncomplicated postoperative recovery and remained well upon routine follow-up.

## 3. Discussion

Adult intussusception is rare compared to intussusception seen in children. Diagnosis can be challenging and often delayed due to the nature of often prolonged, nonspecific symptoms [1]. In contrast, diagnosis in children is readily made using ultrasound which demonstrates the characteristic “target sign” produced by the mesenteric fat of the intussusceptum [2, 3]. Following diagnosis, timely management with air enema yields excellent results with the need for surgical intervention not required routinely [4].

Acute diagnosis of intussusception in adult populations is difficult, with diagnosis beyond intestinal obstruction often not made preoperatively [1, 5]. Plain abdominal XRs are considered the first-line imaging option in diagnosis of intestinal obstruction and may provide some information regarding the obstruction site [1, 6]. However, AXR is not valuable in the diagnosis of intussusception [7]. CT is often the choice modality to investigate prolonged abdominal pain as is often seen in adult intussusception [1, 8, 9]. CT, with characteristic findings of target or sausage-shaped soft tissue mass, has been shown to be superior to other modalities with good diagnostic accuracy and increase preoperative diagnosis [1, 7–9]. In our case, there was early reluctance to investigate with CT given the patient's age. However, with symptoms not progressing and inflammatory markers worsening, CT imaging was able to provide the diagnosis of intussusception preoperatively.

The general consideration of management for adult intussusception is that surgical intervention is required [10]. However, controversy still etches around the extent of bowel resection and the manipulation of the intussuscepted bowel during reduction [7, 11]. Traditional argument advocates for resection in the absence of reduction as adult intussusception has a high association with malignancy [7, 12]. Evolution of management processes now reflects the extent of involved small bowel with extensive involvement undergoing initial reduction to reduce the amount of intestine resected [7, 13]. Proponents of this methodology argue this to be safe as primary malignancy risk in small bowel intussusception is low [14]. It has also ben argued that reduction alone is adequate when there is enteric intussusception with proven benign aetiology and viable tissue [10]. With further argument, proposing that preoperative and intraoperative reduction of intussusception, when in the absence of necrosis, will likely become the standard approach as greater benefits can be offered, including reduction in extent of resection, increased time and preparation to allow for more radical surgery for cancer, and the avoidance of emergency surgery [7].

The management of intussusception secondary to Meckel's diverticulum shares more consensus throughout the literature. Intussusception due to Meckel's diverticulum is a definite indication for diverticulectomy or segmental resection [15, 16]. The bowel should be examined closely for ischaemia, and further resection of the bowel is warranted if ischaemia is present [15, 17, 18]. Noted in the literature are cases of intussusception secondary to Meckel's diverticulum being managed with initial reduction followed by segmental resection and diverticulectomy [1, 15]. This approach likely needs further validation.

## 4. Conclusion

Adult intussusception in adults is a rare and often presents a diagnostic dilemma. An uncommon cause of adult intussusception is Meckel's diverticulum. CT imaging provides good diagnostic accuracy for intussusception [1, 8, 9]. In this case, patient age stood as a barrier to early CT imaging; however, CT was appropriately performed following clinical deterioration of the patient. The management of adult intussusception where Meckel's diverticulum is the aetiology is a clear indication for small bowel resection. The literature acknowledges that there may be a role for initial reduction; however, this approach likely requires further validation [1, 15].
